# Effectiveness of occupational therapy in Parkinson’s disease: study protocol for a randomized controlled trial

**DOI:** 10.1186/1745-6215-14-34

**Published:** 2013-02-02

**Authors:** Ingrid HWM Sturkenboom, Maud J Graff, George F Borm, Eddy MM Adang, Maria WG Nijhuis-van der Sanden, Bastiaan R Bloem, Marten Munneke

**Affiliations:** 1 Nijmegen Centre of Evidence Based Practice, Department of Rehabilitation-Occupational Therapy (898), Radboud University Nijmegen Medical Centre, Reinier Postlaan 2, 6525GC, PO Box 9101, 6500HB, Nijmegen, The Netherlands; 2 Nijmegen Centre of Evidence Based Practice, Scientific Institute for Quality of Healthcare, Radboud University Nijmegen Medical Centre, Nijmegen, The Netherlands; 3 Department of Epidemiology, Biostatistics and HTA, Radboud University Nijmegen Medical Centre, Nijmegen, The Netherlands; 4 Donders Institute for Brain, Cognition and Behavior; Department of Neurology, Radboud University Nijmegen Medical Centre, Nijmegen, The Netherlands; 5 Nijmegen Centre of Evidence Based Practice, Department of Neurology, Radboud University Nijmegen Medical Centre, Nijmegen, The Netherlands

**Keywords:** Parkinson disease, Occupational therapy, Guidelines, caregiver, Randomized controlled trial, Study protocol, Effectiveness, Economic evaluation

## Abstract

**Background:**

Occupational therapists may have an added value in the care of patients with Parkinson’s disease whose daily functioning is compromised, as well as for their immediate caregivers. Evidence for this added value is inconclusive due to a lack of rigorous studies. The aim of this trial is to evaluate the (cost) effectiveness of occupational therapy in improving daily functioning of patients with Parkinson’s disease.

**Methods/Design:**

A multicenter, assessor-blinded, two-armed randomized controlled clinical trial will be conducted, with evaluations at three and six months. One hundred ninety-two home-dwelling patients with Parkinson’s disease and with an occupational therapy indication will be assigned to the experimental group or to the control group (2:1). Patients and their caregivers in the experimental group will receive ten weeks of home-based occupational therapy according to recent Dutch guidelines. The intervention will be delivered by occupational therapists who have been specifically trained to treat patients according to these guidelines. Participants in the control group will not receive occupational therapy during the study period. The primary outcome for the patient is self-perceived daily functioning at three months, assessed with the Canadian Occupational Performance Measure. Secondary patient-related outcomes include: objective performance of daily activities, self-perceived satisfaction with performance in daily activities, participation, impact of fatigue, proactive coping skills, health-related quality of life, overall quality of life, health-related costs, and effectiveness at six months. All outcomes at the caregiver level will be secondary and will include self-perceived burden of care, objective burden of care, proactive coping skills, overall quality of life, and care-related costs. Effectiveness will be evaluated using a covariance analysis of the difference in outcome at three months. An economic evaluation from a societal perspective will be conducted, as well as a process evaluation.

**Discussion:**

This is the first large-scale trial specifically evaluating occupational therapy in Parkinson’s disease. It is expected to generate important new information about the possible added value of occupational therapy on daily functioning of patients with Parkinson’s disease.

**Trial registration:**

Clinicaltrials.gov: NCT01336127.

## Background

Parkinson’s disease is the second most common neurodegenerative disorder. It is a complex disease affecting both motor and non-motor systems in the brain. As a result patients can have a wide range of deficits in performance components, including mobility, balance, hand dexterity, memory and executive functioning. As the disease progresses, effectiveness of the medication regime often decreases, and daily functioning and social participation become increasingly compromised [1-3]. Parkinson’s disease has a great impact on the quality of life of both patients and their informal caregivers [4-6]. The costs of care are high, partly due to the increasing need of support [4]. Improvement of quality of life and reduction of healthcare costs might be achieved by maintaining or improving the patient’s skills and independence in daily activities, and also by reducing caregivers’ burden. To address the great variety of needs in a complex and progressive disease like Parkinson’s disease, a client-centered and multidisciplinary approach is required [7-9].

Within multidisciplinary care for Parkinson patients, the primary role of occupational therapy (OT) is to optimize activity performance and engagement in valued activities and roles in the home or community context (occupational performance). The contribution of OT in Parkinson’s is widely recognized, but systematic reviews reveal a lack of rigorous studies to draw conclusions on the effectiveness of OT in Parkinson’s care [10-12]. Some studies evaluate OT as part of a multidisciplinary intervention [13-17], but the specific contribution and added value of OT cannot be determined from these studies.

From 2006 to 2008 we developed guidelines for OT in Parkinson’s disease (in Dutch), under the auspices of the Dutch Association of Occupational Therapy with the aim to improve uniformity and quality of OT in Parkinson’s disease [18,19]. The guidelines cover specific methods for occupation-based assessment of patients and their caregivers and self-management and compensatory strategies to maintain or enhance occupational performance or occupational performance patterns in daily life.

Our hypothesis is that OT according to the Dutch guidelines has an *added* value within multidisciplinary care for patients with Parkinson’s disease and their caregivers. We expect that addressing the complex occupational performance issues from an OT perspective will improve daily functioning, more so than if OT is not involved. Improved daily functioning will result in enhanced participation in daily activities among patients, reduced caregiver burden, an improved quality of life for both patients and caregivers, and a reduction in costs for society. To test this hypothesis, we followed the steps of the framework for evaluation of complex interventions of the Medical Research Council [20,21]. Based on a phase II exploratory trial [22] we have improved the procedures for the currently proposed randomized controlled trial (phase III trial). This trial, the OTiP study, evaluates the effectiveness and cost-effectiveness of OT according to the Dutch guidelines for OT in Parkinson’s disease.

## Methods/Design

### Trial design

A multicenter, assessor-blinded, two-armed randomized controlled clinical trial will be conducted. Patients and their caregivers will be assigned to the experimental group or to the control group in a ratio of 2:1, respectively. This way the patients have twice as much chance to be in the intervention than in the control group. This ratio will enhance the inclusion, whereas there will hardly be any power loss compared to a 1:1 randomization. Randomization will be based on a computerized minimization algorithm with the following minimization factors: baseline primary outcome measure (Canadian Occupational Performance Measure (COPM) performance: <5; ≥5), severity of disease (Hoehn and Yahr (H&Y) score: <3; ≥3), gender and age group (<65 years; ≥65 years) of the patient, and patient receiving physiotherapy at baseline (yes/no).

**Figure 1:**
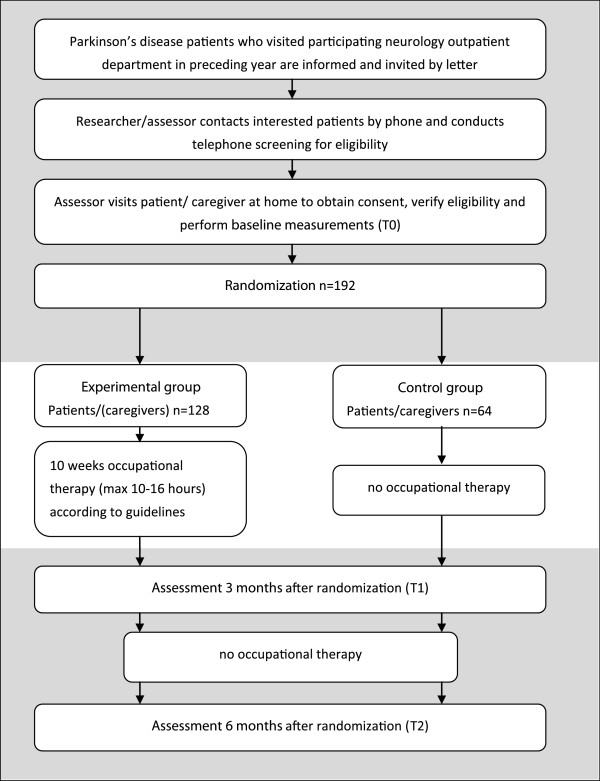
Flow chart of design and enrollment procedures.

Data on observational and self-reported outcome measures will be collected at baseline, after three months (post-intervention) and after six months (follow-up) (see Figure 1).

Full ethical approval has been granted by the medical ethical committee of Arnhem-Nijmegen (NL27905.091.09/ABR27905) and the OTiP trial is registered at clinicaltrials.gov (NCT01336127).

### Setting

For inclusion and intervention the study is embedded within ParkinsonNet regions in the Netherlands. ParkinsonNet comprises 65 regional networks of professionals specialized in the treatment of patients with Parkinson’s disease, and includes a group of specifically trained occupational therapists [23,24]. Ten regional hospitals and 18 occupational therapists in nine selected ParkinsonNet regions agreed to participate. The trial assessments and OT interventions take place at the patient’s home.

### Participants

Eligible patients have idiopathic Parkinson’s disease, live at home, and report difficulties in valued daily activities covering the OT domains of self-care, domestic activities, work or leisure. Exclusion criteria are: OT intervention in the last three months, predominating disabling comorbidity, and inability to complete questionnaires (that is, due to language problems or a Mini Mental State Examination score <24). A primary informal caregiver of each patient can participate in the study when willing and available. Enrollment will take place over a period of 18 months (2011/2012) and procedures are given in Figure 1. Informed consent of patient and caregiver is obtained before the first assessment.

### Intervention

**Figure 2:**
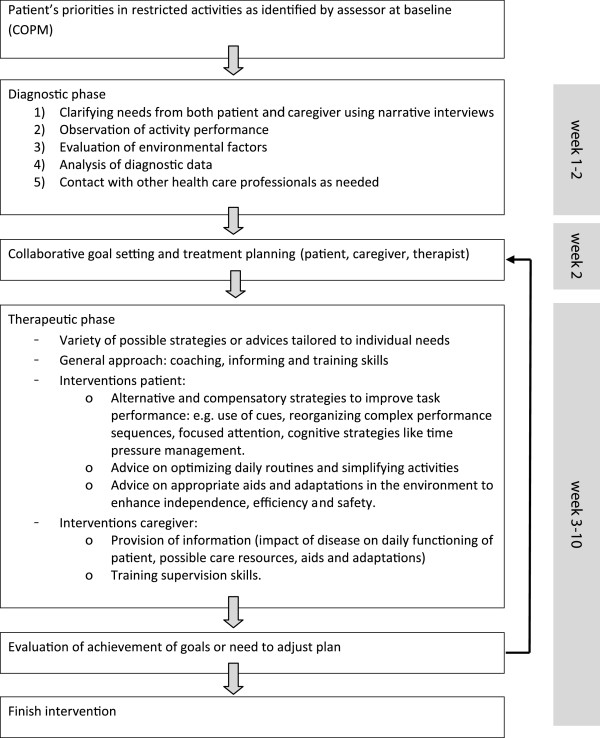
Characteristics and process of the intervention.

The OTiP intervention protocol follows the principles and recommendations for diagnostics and interventions as described in the Dutch guidelines for OT in Parkinson’s disease [18,19]. The approach is client centered including shared decision making and supporting self-management of the patient and caregiver in dealing with problems in daily activities. The trial therapist receives the patient’s priorities in problems in daily functioning as evaluated at baseline with the COPM [25]. The baseline COPM priorities and additional information from the diagnostic phase shape the treatment plan. The intervention is delivered at the patient’s home for a period of ten weeks within three months. Depending on complexity of goals, the amount of sessions can vary with a maximum of ten sessions (only patient goals) or 16 sessions (patient and caregiver goals) of 45 to 60 minutes. Between the three and six month assessments, no OT will be received. An exception is when in incidental cases of lengthy procedures to apply for aids and adaptations, a follow-up contact after delivery is necessary to ensure safe and proper use of the equipment. Figure 2 summarizes the process and characteristics of the OTiP intervention.

The control group does not receive any OT intervention during the study period (six months). Thereafter, control group patients can receive OT according to the OTiP protocol if they wish.

In both groups, patients and caregivers are allowed to receive other medical or allied health care interventions except OT during the study period. We register the input of other health and social care professionals using a care-utilization questionnaire at each of the three assessments focusing on the preceding three months.

### Training of trial therapists

Before the start of the trial, the participating occupational therapists follow a three-day training to inform them about the study procedures and to train them to treat the patients and caregivers according to the OTiP intervention protocol. Special attention is given to enhancing the therapists’ skills in coaching and motivational interviewing and in eliciting and collaboratively defining meaningful, individualized goals with the patient and caregiver. Ways to achieve sufficient treatment intensity in ten weeks are discussed. Halfway through the inclusion period a booster training session (one day) is planned. Therapists can use a secure online platform to share issues and experiences and can consult an expert OT (expertise in the OTiP protocol) to discuss the intervention.

### Assessment procedures

Data from patients and caregivers will be collected at baseline (T0), three months (T1) and six months (T2) by three research assistants (see Table 1). Another eight selected and trained occupational therapists will score the Perceive Recall Plan Perform system (PRPP [26]) in an activity that is video recorded by the assessor. All assessors and PRPP-scorers are blinded for group allocation and each participant will be followed up by the same assessor. Patients and caregivers also fill in self-report questionnaires. Observational tests or measures that follow a semi-structured interview format are conducted in the patient’s home environment by the assessor. Considering possible response fluctuations in Parkinson’s, measures are administered within one to two hours after medication intake (the *on* phase). For budgetary reasons, the six month assessment is conducted by phone and therefore, does not include observational measures.

**Table 1:** Outcome measures

**Participant**	**Outcome measure**	**Instrument**	**baseline**	**3 months**	**6 months**
**Patient**	Self-perceived performance in daily activities^a^	Canadian Occupational Performance Measure (COPM; performance rating) [25]	*√*	*√*	*√*
	Self-perceived satisfaction with performance in daily activities	Canadian Occupational Performance Measure (COPM; satisfaction rating) [25]	*√*	*√*	*√*
	Objective performance in daily activities	Perceive Recall Plan Perform system (PRPP) [26]	*√*	*√*	-
	Participation	Activity Card Sort (ACS) [27,28]	*√*	*√*	-
		Utrecht Scale for Evaluation of Rehabilitation Participation (USER-P; satisfaction part) [29,30]	*√*	*√*	*√*
	Health-related quality of life	Parkinson’s Disease Questionnaire (PDQ-39) [31-33]	*√*	*√*	*√*
	Impact of fatigue	Fatigue Severity Scale (FSS) [34,35]	*√*	*√*	*√*
	Mood	Becks Depression Inventory (BDI) [36]	*√*	*√*	*√*
**Caregiver**	Perceived caregiver burden	Zarit Burden Interview (ZBI) [37]	*√*	*√*	*√*
	Objective caregiver burden	Objective care burden questionnaire; hours of care	*√*	*√*	*√*
	Mood	Hospital Anxiety and Depression Scale (HADS) [38]	*√*	*√*	*√*
**Both**	Quality of life	Euroqol EQ-5 D [39]	*√*	-	*√*
	Quality of life overall	Visual Analogue Scale for Quality of life; VAS QoL	*√*	*√*	*√*
	Proactive coping	Utrecht Proactive Coping Competence scale (UPCC) [40]	*√*	*√*	*√*
	Resource utilization	Resource utilization questionnaire: patient and caregiver version	*√*	*√*	*√*

^a^Primary outcome measure.

### Outcome measures

#### Primary outcome measure

The primary outcome for effectiveness of this intervention is the patient’s self-perceived performance in daily functioning as assessed with the Canadian Occupational Performance Measure (COPM) (see Table 1) [25]. The COPM is an individualized outcome measure with a semi-structured interview format and a structured scoring method. The patient identifies three to five activities in which he encounters problems and would like to improve. These activities are subsequently rated by the patient on a scale from 1 to 10 for perceived performance capacity and level of satisfaction with this. The COPM score for performance or satisfaction derives from the mean score of the prioritized activities. Change is evaluated by asking the patient to rescore performance and satisfaction on the original priorities. Studies evaluating the psychometric properties of the COPM (in populations of stroke and various chronic conditions) support the validity and reliability of the COPM [41-43]. Responsiveness for change over three months was established in a population with various conditions, and the results support both criterion and construct responsiveness [44].There is a high correlation between performance and satisfaction scores.

We selected the COPM as a primary outcome measure in our trial as it fits with the client-centered nature and specific focus of the OTiP intervention. It addresses the patient’s priorities and evaluation of valued activities. In the assessment procedures, we specified the COPM administration protocol to improve uniformity in the semi-structured interview and in the formulation of priorities while taking care to maintain the client-centered nature of the instrument. Only the mean performance capacity score will be used as primary outcome.

#### Secondary outcome measures

In Table 1 all secondary outcome measures are listed. Patients’ secondary outcomes include evaluation of observed performance of daily activities, self-perceived satisfaction with performance in daily activities, participation, impact of fatigue, proactive coping skills, mood, health related quality of life, and overall quality of life. All outcomes at the caregiver level will be secondary and include self-perceived burden of care, objective burden of care, proactive coping skills, mood and overall quality of life. In both patients and caregivers resource use, productivity losses and other costs related to receiving support or providing care are assessed with a questionnaire for the economic evaluation

#### Background variables

Socio-demographic data consisting of age, gender, marital status, education, employment status, and relationship between client and caregiver will be collected at baseline, using a questionnaire. Patient’s disease severity will be measured with the Unified Parkinson Disease Rating Scale-part III [45] and with the Hoehn and Yahr (H&Y) scale. The Mini Mental State Examination [46] is used for cognitive screening. Comorbidity is checked with an open question for screening purposes.

#### Process measures

To enable explanation of results, process data will be collected. Therapists complete standardized OTiP patient records and a process evaluation summary sheet to provide insight in adherence to the steps of the protocol and actual treatment delivery (content, amount of sessions and time spent). For each patient seen, the participating trial therapists also record their views of effectiveness of the intervention for the individual patient and caregiver on the process evaluation summary sheet. We will compare priorities identified by patients in the baseline COPM, with goals addressed in the intervention. At the end of the study a focus group will be conducted with all therapists exploring their experiences and views on conducting the OTiP intervention protocol in daily practice.

Patients and caregivers experiences with the intervention will be evaluated with a custom made questionnaire (OTiP-CQ questionnaire) based on the Consumer Quality index [47,48]. It includes mainly closed questions on experiences with the interaction with the therapist, the process and content of the intervention and the perceived effectiveness of the intervention. Assessors register any irregularities in adherence to assessment procedures, including unblinding. Adverse events or irregularities affecting protocol adherence will be registered by the researcher.

### Sample size calculation

In the main study, we intend to enroll 192 patients with Parkinson’s and their caregivers. This is based on the results of the OTiP pilot study with 43 participants. The pilot resulted in a difference of 0.5 in scores on the primary outcome measure (COPM), whereas the standard deviation was 1.35. Based on these assumptions, a *t*-test would require a control group of 75 patients and an intervention group of 150 patients for 80% power (two-sided testing at 5%). The correlation between baseline and outcome was approximately 0.5 and as a result, the co-variance analysis that is planned only requires a control group of 56 and an intervention group of 112 patients (total of 168) [49]. In the feasibility study the dropout rate was 7%. We expect however, that dropout rates will be higher in this main study as the study period is doubled. Therefore we adjust for a 10 to 15% drop- out rate and will include approximately 192 patients.

### Statistical analysis

#### Descriptive statistics

Means, standard deviations and frequencies will be used to describe outcome, background and baseline variables.

#### Analysis effectiveness

The primary variable for effectiveness will be analyzed in a covariance model with the COPM scores after three months (T1) as dependent variable. The baseline COPM scores (T0) and the minimization factors will be covariates. Two-sided 95% confidence intervals will be calculated. The analysis follows the principle of intention to treat. Similarly, a secondary analysis will be done evaluating the secondary outcome variables and outcomes for six months. Regarding the caregiver outcomes, we plan a subanalysis for caregivers with low perceived burden at baseline (Zarit Burden Interview (ZBI) ≤20) and high perceived burden of care (ZBI >20).

#### Analysis cost-effectiveness

An economic evaluation will be done from a societal perspective by evaluating the differences in total costs in the control and experimental group at three and six months. Total costs include care consumption and productivity loss of patients and caregivers related to Parkinson’s and caregiver’s hours of care provision to the patient. The number of OT sessions and total time spent on OT, will be translated as direct costs of the intervention. Differences in costs between groups over a six-month timeframe will be estimated using regression analysis taking into account potential co-variants. Secondly, utility will be calculated as quality adjusted life year (QALY) over a timeframe of six months using the trapezium rule. QALYs for patients and caregivers are derived from the EuroQol EQ-5D scores using the EQ-5D health tariffs for the Dutch population [50]. Then, cost and QALY differences are combined in an incremental cost-effectiveness ratio (ICER), and using the bootstrap method, confidence intervals surrounding this ICER will be estimated. We also measure cost-effectiveness by costs per successful treatment. A successful treatment is a treatment with a clinical relevant positive change in the COPM (+ 2 points) at six months. Reporting the ICER as cost per successful treatment may provide decision-makers with a relatively intuitive means of assessing cost-effectiveness, because the denominator of the incremental ratio is calculated using a clinically meaningful objective.

#### Analysis process data

A descriptive analysis will be performed for the quantitative data on the evaluation forms of participants and assessors and the data of therapists of the given intervention. We will analyze the data from the focus group discussion following the constant comparative method [51].

## Discussion

Current evidence for the effectiveness of OT in Parkinson’s disease is scarce and inconclusive. The OTiP trial is the first large-scale randomized control trial evaluating the effectiveness and cost-effectiveness of client-centered OT in Parkinson’s disease.

It is difficult to select one comprehensive outcome measure to reflect the effectiveness of a complex intervention that has a broad and individualized scope. We have chosen an outcome measure (namely the COPM) that potentially fits best with the client-centered nature of the OTiP intervention. Additional (secondary) outcomes can be used to capture the multimodal nature of the intervention. For this purpose, we have included a range of secondary outcome measures covering the wide scope of the OTiP intervention.

The main inclusion criteria are self-perceived problems in valued daily activities and the extent to which patients perceive limitations in daily activities or participation restrictions. These inclusion criteria do not always correspond with disease severity or factors like age. Therefore, we expect great diversity in characteristics of our participants and their contexts. This might also result in diversity in outcomes.

To cover the large geographical spread of participants in this multicenter trial, a relatively high number of trial therapists will be involved to deliver the intervention. This means that the average number of patients seen by each therapist within the trial is low. We have taken several measures to enhance and monitor protocol adherence, including an online discussion platform and opportunities for coaching during the study.

Recruitment for trials is often difficult. A strength of this trial is the presence of the national ParkinsonNet infrastructure within the Netherlands [23,24]. This allows easier access to neurologists in the participating regional and university hospitals and their pool of patients. Another important and novel aspect in this study is that all other interventions are allowed to take place during the study. This way, the added value of guideline-based OT in a usual multidisciplinary care setting can be evaluated. With the comprehensive process evaluation it will also provide information on factors that are important for further improvement of the content or implementation of the guidelines.

## Trial status

The status of the trial is ongoing at the time of manuscript submission. The recruitment of participants is expected to be completed by November 2012.

## Abbreviations

COPM: Canadian Occupational Performance Measure; H&Y: Hoehn and Yahr scale; ICER: incremental cost-effectiveness ratio; OT: occupational therapy; PRPP: Perceive Recall Plan Perform system; QALY: quality adjusted life year; ZBI: Zarit Burden Interview

## Competing interests

The authors declare that they have no financial or non-financial competing interests in relation with this manuscript.

## Authors’ contributions

MM, BB, IS, MN, MG, GB, and EA contributed to the research design. MM, BB, and IS acquired funding for the trial. IS wrote the first draft of the manuscript and was responsible for revisions. All authors read and approved the final manuscript.
